# A Comparison of Seizure Prophylaxis: Phenytoin Versus Levetiracetam

**DOI:** 10.7759/cureus.14956

**Published:** 2021-05-11

**Authors:** Brian Fiani, Christopher Andraos, Iveth Mabry, Javed Siddiqi

**Affiliations:** 1 Neurosurgery, Desert Regional Medical Center, Palm Springs, USA; 2 College of Natural and Agricultural Sciences, University of California Riverside, Riverside, USA; 3 Pharmacy, Arrowhead Regional Medical Center, Colton, USA; 4 Neurosurgery, Riverside University Health System Medical Center, Moreno Valley, USA; 5 Neurosurgery, Arrowhead Regional Medical Center, Colton, USA; 6 Neurosurgery, California University of Science and Medicine, Colton, USA

**Keywords:** seizure prevention, general pharmacology, pharmacology and therapeutics, seizure prophylaxis, seizure risk

## Abstract

Phenytoin and levetiracetam are both antiepileptic drugs (AEDs) used for seizure prophylaxis. However, to date, there is a paucity of literature comparing their relative efficacies. In this narrative review, we seek to determine if there is greater advantage between the two AEDs, levetiracetam and phenytoin. Phenytoin is the more traditional AED of the two as it has been medically used for a much longer time than levetiracetam. However, levetiracetam, the newer AED of the two, has fewer side effects than phenytoin and fewer drug-drug interactions. Although past studies have aimed to compare the efficacy of phenytoin versus levetiracetam, there is no clear consensus as to if there is a clinical advantage to one over the other. Here, we have analyzed several studies published between 2013 and 2020 in the hopes of having a better understanding of which AED is more efficient in preventing seizures. Many factors can contribute to determining which AED is the better fit for patients, including pricing, risk for adverse drug effects, and level of patient monitoring. After analysis of past research, the more advantageous AED still remains unclear. Future research must be conducted that involve large patient populations, stratifying age populations, and studies analyzing cost-effectiveness to clearly determine if there is indeed a more advantageous AED between levetiracetam and phenytoin.

## Introduction and background

Levetiracetam and phenytoin are two of the most commonly used antiepileptic drugs (AEDs) in seizure prevention. However, there is little data on their comparative efficacies. Prolonged seizures that may occur after traumatic brain injury (TBI), intracranial hemorrhage, or neoplastic augmentation of cortical fibers are capable of causing permanent neurological deficits by causing neuronal cell death. Isolated and short seizures are less likely to cause detrimental changes in brain function but may still cause possible loss of brain cells. Hence, there is great importance for seizure prevention through the use of AEDs. Phenytoin was approved for medical use in 1953, whereas levetiracetam was approved for medical use in 1999 [1]. Historically, phenytoin has been the most prescribed medication for seizure prophylaxis. On the other hand, levetiracetam is a newer AED with much fewer side effects than phenytoin, fewer drug-drug interactions, while also having a straightforward dosing regimen [2]. Although phenytoin tends to be effective in most settings, it also has an elevated side effect frequency and is largely impacted by certain other medications [3].

Recently, the use of levetiracetam for seizure prophylaxis has risen. Levetiracetam’s simple dosing and the lack of drug level monitoring contributes to the rise in its use. Phenytoin has also been linked with a number of adverse side effects, including cutaneous hypersensitivity and CYP-450 induction, which are not seen with levetiracetam [4]. Currently, there is no clear consensus as to which AED is superior in the prevention of seizures or whether there is any clear winner between phenytoin and levetiracetam. Here, we review the literature to date regarding the comparison in the use of phenytoin and levetiracetam to best determine whether there is a consensus of preference for antiepileptic usage.

## Review

Pharmacology

Mechanisms of Action

Phenytoin is an AED that works by binding and stabilizing the inactivated state of sodium channels and blocks the high-frequency firing of neurons and spread of seizure activity [5]. Phenytoin is highly protein-bound and is metabolized by cytochrome P450 (CYP) enzymes, in addition to being a CYP inducer [6]. On the other hand, although the exact mechanism of levetiracetam is largely unknown, it has been shown to enhance gamma aminobutyric acid (GABA) activity and partially inhibit voltage-dependent N-type calcium channels [7].

Indications

Phenytoin has multiple indications, including control of generalized tonic-clonic and complex partial seizures, in addition to the prevention and treatment of seizures occurring during or following neurosurgery [5]. Additionally, phenytoin is used in the treatment of status epilepticus and, off-label, as prophylaxis of early post-traumatic seizure in TBI [4,8,9].

Levetiracetam is indicated as an adjunct therapy in the treatment of seizures such as partial-onset seizures in adults and children aged four years and older with epilepsy, myoclonic seizures in adults, and adolescents aged 12 years and older with juvenile myoclonic epilepsy, as well as myoclonic seizures in adults and adolescents aged 12 years and older with juvenile myoclonic epilepsy [7]. Off-label, levetiracetam is used in the treatment of status epilepticus as short-term seizure prophylaxis in subarachnoid hemorrhage and severe acute post-TBI [10,11].

Contraindications

Phenytoin is contraindicated in patients with a history of hypersensitivity to phenytoin, other hydantoins, or any inactive ingredient in phenytoin [5]. There are additional contraindications with injectable phenytoin such as sinus bradycardia, sinoatrial block, second- and third-degree heart block, and Adams-Stokes syndrome [6]. Similarly, the use of levetiracetam is contraindicated in patients who have a history of hypersensitivity to levetiracetam or any inactive ingredients in levetiracetam [7].

Trials and Outcomes

A retrospective observational study conducted by Kruer et al. evaluated seizure incidence seven days after TBI in patients treated with phenytoin and levetiracetam, as well as characterized the practice of AED selection [12] (Table 1). Patients were excluded from the study if they were younger than 18. Of 109 patients, 89 received phenytoin while 20 received levetiracetam. In total, two patients experienced posttraumatic seizure, one in each group. Kruer et al,. concluded that after the approval of intravenous levetiracetam, a trend favoring levetiracetam for seizure prevention was observed [12]. A separate retrospective cohort study was conducted by Radic et al. to compare the efficacy and risk of using levetiracetam versus phenytoin for seizure prophylaxis following acute or subacute subdural hematoma diagnosis [2]. A total of 124 patients were placed in the phenytoin group while 164 patients were placed in the levetiracetam group. It was found that there was no significant difference in clinical and/or electrographic seizure risk, though there was a decreased risk of adverse events in the levetiracetam group. In subjects with a midline shift of >0 mm, levetiracetam was associated with an increased risk of electrographic seizures during hospitalization and a decreased risk of adverse drug effects when compared with phenytoin uses. Radic et al. concluded that levetiracetam is associated with a lower risk of adverse drug effects.

**Table 1:** Comparison studies of phenytoin and levetiracetam. TBI: traumatic brain injury; AED: antiepileptic drugs; CSE: convulsive status epilepticus; ARS: acute repetitive seizures

Author, year	Objective	Outcome
Kruer et al., 2013 [12]	Evaluated seizure incidence seven days after TBI in patients treated with phenytoin versus levetiracetam	Only two out of 109 patients experienced posttraumatic seizures after receiving AED, indicating low incidence. After the approval of intravenous levetiracetam, a trend favoring the use of levetiracetam was observed
Inaba et al., 2013 [4]	Compared efficacy of phenytoin with that of levetiracetam for preventing early posttraumatic seizures	There was no significant difference in levetiracetam and phenytoin efficacy. Seizure rates for both AEDs were 1.5%. Therefore, the cost and need for serum monitoring should be considered in guiding the choice of prophylactic agents
Radic et al., 2014 [2]	Compared the efficacy and risk of using levetiracetam versus phenytoin for seizure prophylaxis following acute or subacute subdural hematoma diagnosis	Levetiracetam had a similar efficacy to phenytoin in preventing clinical and/or electrographic seizures following acute/subacute subdural hematoma diagnosis. Patients with a midline shift of >0 mm have higher risk of electrographic seizures when treated with levetiracetam. Levetiracetam is associated with a lower risk of adverse drug effects
Chakravarthi et al., 2015 [13]	Compared the safety and efficacy of intravenous levetiracetam with intravenous phenytoin in the management of status epilepticus	Phenytoin achieved control of status epilepticus in 15 of 44 patients. Levetiracetam achieved control of status epilepticus in 13 of 44 patients. Levetiracetam is an attractive and effective alternative to phenytoin in management of status epilepticus
Khan et al., 2016 [14]	Compared the efficacy of phenytoin and levetiracetam in the prevention of early posttraumatic seizures in moderate-to-severe TBI	Levetiracetam effectively controlled seizures in 73 out of 77 patients. Phenytoin controlled seizures in 70 out of 77 patients. There is no statistically significant difference in the efficacy of phenytoin and levetiracetam in prophylaxis of early posttraumatic seizures in cases of moderate-to-severe TBI
Singh et al., 2018 [15]	Compared the efficacy of intravenous phenytoin and intravenous levetiracetam in acute seizures	Of the 100 children in this study, three in levetiracetam and two in phenytoin group had a repeat seizure in 24 hours. Intravenous levetiracetam and phenytoin have similar efficacy in preventing seizure recurrences for 24 hours in children aged 3-12 years presenting with acute seizures
Zhao et al., 2018 [16]	Evaluated the safety and efficacy of levetiracetam in the prevention of brain traumatic seizures with the standard drug phenytoin	Levetiracetam is more effective than phenytoin in preventing early seizures. There is no statistically significant difference of phenytoin and levetiracetam in the prevention of overall and late seizures
Noureen et al., 2019 [17]	Compared the efficacy and safety of levetiracetam and phenytoin as a second-line anticonvulsant for the management of childhood convulsive status epilepticus	Intravenous levetiracetam is significantly more effective than intravenous phenytoin as a second-line drug for treating CSE in children who have failed to respond to benzodiazepines. Treatment efficacies of levetiracetam versus phenytoin were 278/300 (92.7%) and 259/300 (83.3%), respectively
Lyttle et al., 2019 [18]	Compared the efficacy and safety of phenytoin and levetiracetam for second-line management of pediatric convulsive status epilepticus	Convulsive status epilepticus was terminated in 106/152 (70%) in the levetiracetam group compared to 86/134 (64%) in the phenytoin group. Although levetiracetam was not significantly superior to phenytoin, the results along with the previously reported safety profiles and its comparative ease of administration of levetiracetam suggest it is an appropriate alternative to phenytoin as the first choice second-line anticonvulsant in the treatment of pediatric convulsive status epilepticus
Besli et al., 2020 [19]	Compared the efficacy and safety profile of intravenous levetiracetam and phenytoin as second-line agents in children with convulsive status epilepticus and acute repetitive seizures	Intravenous levetiracetam was as effective as intravenous phenytoin in emergency treatment of children with ARS (55.8% vs. 58.8%, respectively). Also, levetiracetam is more effective than phenytoin in treatment of CSE (77.6% vs. 57.7%, respectively). Intravenous levetiracetam is a favorable option as a first second-line AED for pediatric seizures

Chakravarthi et al. aimed to compare the safety and efficacy of intravenous levetiracetam with intravenous phenytoin in the management of status epilepticus [13]. In this study, 44 patients were randomized to receive either phenytoin or levetiracetam. The primary endpoint was successful clinical termination of seizure activity within 30 minutes of drug administration. Secondary endpoints were the recurrence of seizures within 24 hours, drug-related adverse effects, mortality during hospitalization, and need for ventilatory assistance. Phenytoin achieved control of status epilepticus in 15 patients compared to levetiracetam in 13 patients. This study concluded that levetiracetam is as effective as phenytoin in respect to the outcome measures. Levetiracetam is favored due to its relative ease of administration and lack of continuous monitoring. Levetiracetam is an appealing alternative to phenytoin in management of status epilepticus. In a separate randomized controlled trial conducted by Khan et al., the authors aimed to compare the efficacy of phenytoin and levetiracetam in the prevention of early posttraumatic seizures in moderate-to-severe TBI [14]. The 154 patients in this study were equally divided into two groups. Phenytoin was effective in preventing posttraumatic seizures in 73 patients whereas levetiracetam effectively controlled seizures in 70 cases. The study concluded that there is no statistically significant difference in the efficacy of phenytoin and levetiracetam in prophylaxis of early posttraumatic seizures in cases of moderate-to-severe TBI.

Noureen et al. aimed to compare the clinical efficacy and safety of intravenous levetiracetam versus intravenous phenytoin as second-line drugs in the management of status epilepticus in children [17]. In this open-label, randomized controlled trial, 300 children with status epilepticus received levetiracetam and another 300 children with status epilepticus received phenytoin. Levetiracetam was effective in 278/300 cases while phenytoin was effective in preventing seizures in 259/300 cases. Further, adverse events were observed in eight children in the phenytoin group. The study concluded that levetiracetam is significantly more effective than phenytoin for the treatment of status epilepticus in children. In a very recent study conducted by Besli et al., the authors aimed to compare the efficacy and safety profile of levetiracetam and phenytoin as second-line treatment agents in children with convulsive status epilepticus and acute repetitive seizures [19]. Out of 227 patients, 141 received levetiracetam while 86 received phenytoin. Levetiracetam was effective in 77.6% of cases while phenytoin was effective in 57.7% of cases in children with convulsive status epilepticus. However, there was no significant difference between the efficacy rates of levetiracetam and phenytoin for acute repetitive seizures (55.8% vs. 58.8%, respectively). The study concluded that levetiracetam seems as effective as phenytoin in treatment of children with acute repetitive seizures, but is more effective for treating convulsive status epilepticus in children. Levetiracetam is a favorable treatment for pediatric seizures.

Future directions

Considering the different studies comparing levetiracetam and phenytoin, there is no clear consensus as to which drug is the more effective AED. Future research, when examining the efficacy of levetiracetam versus phenytoin, should require very large groups with hundreds or even thousands of patients. Moreover, another interesting point to be made is that phenytoin is significantly cheaper than its counterpart levetiracetam. It would be favorable for future trials to include a cost-effectiveness analysis between these two AEDs and evaluate the influence of indirect costs to overall decision-making. More large, randomized, and controlled trials evaluating the safety, quality of life, and effectiveness of these two AEDs are needed. Furthermore, testing the efficacy of levetiracetam versus phenytoin across different age populations may be a future experimental design. When it comes to clinical practice, levetiracetam and phenytoin are both reliable AEDs for treating seizures; however, the level of patient monitoring, risk for adverse drug effects, and pricing should be considered when treatment is administered.

## Conclusions

AEDs are of critical importance in the prevention of seizures, which can permanently injure the brain via neuronal cell death. Levetiracetam and phenytoin are two of the most commonly used AEDs in seizure prevention. Levetiracetam is the newer AED and has much fewer side effects than its counterpart phenytoin. Recently, levetiracetam’s usage for seizure prophylaxis has increased partially due to its lack of need for drug level monitoring and its simple dosing. Phenytoin works by binding to and stabilizing the inactivated state of sodium channels and therein blocks the high-frequency firing of neurons and spread of seizure activity. Levetiracetam’s mechanism of action is largely unknown but has been shown to enhance GABA activity. After reviewing several studies examining the efficacy of phenytoin versus levetiracetam in preventing seizures, there is no definite consensus as to which drug is the more effective AED. More studies must be conducted if we want to determine the equivalency of levetiracetam versus phenytoin. In the meantime, pricing, level of patient monitoring, and risk for adverse drug effects should be evaluated to decide which AED is the better fit for the patient.
